# Função pulmonar em longo prazo após fusão espinhal anteroposterior combinada para escoliose em neurofibromatose tipo 1 e síndrome de Marfan com acompanhamento médio de 13 anos

**DOI:** 10.1055/s-0045-1809526

**Published:** 2025-07-06

**Authors:** José Alberto Alves Oliveira, Rogério dos Reis Visconti, Gustavo Borges Laurindo de Azevedo, Alderico Girão Campos de Barros, Luis E. Carelli, José Roberto Lapa e Silva

**Affiliations:** 1 Faculdade de Medicina, Universidade Federal do Rio de Janeiro, Rio de Janeiro, RJ, Brasil; 2 Instituto Nacional de Traumatologia e Ortopedia Jamil Haddad, Rio de Janeiro, RJ, Brasil

**Keywords:** escoliose, função pulmonar, fusão espinal, neurofibromatose tipo 1, síndrome de Marfan, Marfan syndrome, neurofibromatosis type 1, pulmonary function, scoliosis, spinal fusion

## Abstract

**Objetivo:**

Comparar o efeito da fusão espinhal combinada (anteroposterior) na função pulmonar em pacientes com escoliose secundária à síndrome de Marfan ou à neurofibromatose tipo 1 (NF1) no acompanhamento em longo prazo (> 10 anos).

**Métodos:**

Estudo retrospectivo comparativo com nove pacientes, operados entre março de 1997 a dezembro de 2009. Os pacientes foram divididos em grupos com síndrome de Marfan ou NF1. As medidas de desfecho incluíram sexo, idade (ao diagnóstico e à cirurgia), altura corrigida pela envergadura, índice de massa corporal (IMC), duração da cirurgia (minutos), perda sanguínea estimada (mL), último acompanhamento (anos), complicações pulmonares e relacionadas aos implantes, ângulo de Cobb pré e pós-operatório da curva torácica principal e da cifose torácica (T5 a T12), número de níveis instrumentados, valores absolutos e percentuais previstos de capacidade vital forçada ( *forced vital capacity* , FVC, em inglês) e volume expiratório forçado em um segundo ( *forced expiratory volume in 1 second* , FEV1, em inglês). Os dados foram processados no programa IBM SPSS Statistics for Windows (IBM Corp.), versão 20.0, e as comparações de médias empregaram o teste *t* de Student e a análise de variância ( *analysis of variance* , ANOVA, em inglês), ou os testes de Mann-Whitney e Kruskal-Wallis/Dunn com valor de *p* de 0,05.

**Resultados:**

Não houve diferença nos valores percentuais e absolutos previstos da função pulmonar, ou seja, FVC e FEV1, e no ângulo de Cobb da curva torácica principal entre os grupos nos períodos pré e pós-operatório ( *p* > 0,05). Entretanto, houve redução significativa do ângulo de Cobb da curva torácica principal no grupo com síndrome de Marfan (74→46°; *p* < 0,05).

**Conclusão:**

Não houve piora da função pulmonar nos pacientes submetidos à abordagem combinada após um acompanhamento de mais de 10 anos. Além disso, não houve diferenças significativas nos valores pós-operatórios dos testes de função pulmonar entre os grupos.

Nível de evidência IV; série de casos.

## Abstract (en)

**Objective:**

To compare the effect of combined spinal fusion (anteroposterior) on pulmonary function in patients with scoliosis secondary to Marfan syndrome versus neurofibromatosis type 1 (NF1) at long-term follow-up (> 10 years).

**Methods:**

Retrospective comparative study with nine patients, operated from March 1997 to December 2009, groups: Marfan syndrome versus NF1. Outcome measures were sex; age (at diagnosis and surgery); corrected height by wingspan; body mass index (BMI); duration of surgery (minutes); estimated blood loss (mL); last follow-up (years); pulmonary and implants related complications; pre- and postoperative Cobb angle of main thoracic curve and of thoracic kyphosis (T5 to T12); number of instrumented levels; absolute and percentage predicted values of forced vital capacity (FVC) and forced expiratory volume in 1 second (FEV1). The data was processed in the IBM SPSS Statistics for Windows software (IBM Corp.), version 20.0, and the comparisons of means used the Student's *t* -test and analysis of variance (ANOVA), or the Mann-Whitney and Kruskal-Wallis/Dunn tests, with a *p* -value of 0.05.

**Results:**

There was no difference in the absolute and predicted percentage values of pulmonary function, FVC and FEV1, and Cobb of the main thoracic curve between the groups, pre- and postoperatively ( *p* > 0.05). However, there was a significant reduction in the Cobb of the main thoracic curve in the Marfan syndrome group (74→46°, *p* < 0.05).

**Conclusion:**

There was no worsening of pulmonary function in patients who underwent the combined approach after a follow-up of more than 10 years, and there were no significant differences in the postoperative values of the pulmonary function test between the groups.

Level of evidence IV; case series.

## Introdução

A escoliose afeta cerca de 62% dos pacientes com síndrome de Marfan e é a deformidade da coluna vertebral mais comum. 1 2 Pacientes com deformidade moderada ou grave da caixa torácica, seja por escoliose e/ou pectus excavatum tendem a apresentar um distúrbio restritivo da função pulmonar. 3 4

Na neurofibromatose tipo 1 (NF1) ou doença de Von Recklinghausen, a prevalência de escoliose é de 10 a 60% e a deformidade pode ser distrófica ou não. Nos casos distróficos, pode ocorrer comprometimento significativo da função pulmonar quando há presença de três ou mais características displásicas, sendo elas curva torácica curta, acentuada e única, diminuição da largura das costelas ( *rib pencilling* ), cunhagem de um ou mais corpos vertebrais, massas em tecidos moles paraespinhais ou intraespinhais, rotação acentuada no ápice da deformidade, displasia de pedículos e/ou aumento do canal espinhal e do forame. 5

O tratamento cirúrgico dessas deformidades da coluna na escoliose secundária à NF1 pode ser uma artrodese combinada (anterior e posterior) com a utilização de enxerto autólogo retirado das costelas ressectadas, corrigindo a deformidade com instrumentação estável e maior área cirúrgica para redução dos efeitos da erosão óssea contínua nos casos distróficos. 5 6 Na síndrome de Marfan, as curvas tendem a ser rígidas e de rápida progressão, o que indica a necessidade de intervenção cirúrgica. 7 8

Devido à escassa literatura sobre a avaliação da função pulmonar no acompanhamento em longo prazo após cirurgia para correção da escoliose sindrômica, o presente estudo teve como objetivo comparar o efeito em longo prazo (> 10 anos) da fusão espinhal combinada na função pulmonar em pacientes com escoliose secundária à síndrome de Marfan ou NF1.

## Materiais e métodos

Trata-se de um estudo de série de casos, realizado em um centro de referência nacional em cirurgia da coluna, com avaliação da função pulmonar de 168 pacientes portadores de escoliose operados entre março de 1997 e dezembro de 2009. Os critérios de inclusão foram pacientes de ambos os sexos portadores de escoliose secundária à síndrome de Marfan ou NF1 submetidos à artrodese combinada, com ganchos e parafusos pediculares ou somente implantes de parafusos pediculares, sem toracoplastia e/ou tração halo.

Os critérios de exclusão foram pacientes com cardiopatia, doenças pulmonares, infecção, alterações cognitivas que influenciassem na compreensão dos testes e incapacidade de realizar a avaliação proposta. Os pacientes elegíveis foram submetidos à avaliação durante o último período de acompanhamento com exames de radiografia e espirometria e alocados em dois grupos para comparação: NF1 ou síndrome de Marfan.

Os dados foram obtidos de prontuários médicos e consultas de acompanhamento após aprovação pelo comitê de ética em pesquisa institucional e assinatura do termo de consentimento livre e esclarecido pelos pacientes. Durante o acompanhamento, foram realizados testes de função pulmonar (TFPs) e radiografias (em projeção anteroposterior e perfil), que foram revistos por dois médicos independentes não participantes do estudo, utilizando as mesmas vértebras terminais empregadas no cálculo do ângulo de Cobb pré-operatório. Foram coletados dados das seguintes variáveis: sexo, idade (ao diagnóstico, à cirurgia e ao último acompanhamento), altura corrigida pela envergadura, índice de massa corporal (IMC), duração da cirurgia (minutos), perda sanguínea estimada (mL), acompanhamento (anos), complicações pulmonares e relacionadas ao implante, ângulo de Cobb pré- e pós-operatório da curva torácica principal e cifose torácica (T5 a T12), número de níveis fundidos, valores percentuais e absolutos previstos de capacidade vital forçada ( *forced vital capacity* , FVC, em inglês) e volume expiratório forçado em um segundo ( *forced expiratory volume in 1 second* , FEV1, em inglês).

Os testes de função pulmonar foram realizados com o paciente sentado, usando as manobras de expiração máxima forçada. Nenhum paciente relatou ser fumante. Foram obtidas pelo menos três curvas aceitáveis e duas curvas reprodutíveis. Os seguintes parâmetros foram registrados e classificados de acordo com as diretrizes da American Thoracic Society (ATS) e European Respiratory Society (ERS) 2005. 9

Os diagnósticos de síndrome de Marfan seguiram os critérios de Ghent, 10 que foram atualizados em 2010. 11 Entretanto, em nosso estudo, foram utilizados os critérios vigentes na época. O diagnóstico da NF1 é baseado em critérios clínicos, sendo necessários pelo menos dois critérios. A escoliose distrófica nestes pacientes apresenta-se com uma única curva torácica rígida (< 30% de flexibilidade), curta e acentuada, lapidação das costelas, cunhagem de um ou mais corpos vertebrais, massas em tecidos paravertebrais ou intravertebrais, rotação acentuada no ápice da deformidade, displasia de pedículos; e aumento do canal vertebral e forame. 5 12

A escolha da abordagem combinada foi baseada em critérios radiográficos (curvas rígidas, flexibilidade < 30% e/ou curvas graves, > 80°). Após a fusão intersomática anterior, o paciente permaneceu internado na unidade de terapia intensiva e/ou enfermaria para ser submetido à fusão posterior. À abordagem posterior, osteotomias da coluna posterior ( *posterior column osteotomies* , PCOs, em inglês) foram realizadas em três a quatro níveis. Em todas as cirurgias, foi realizado o teste de despertar, pois, nesse período, a avaliação neurofisiológica intraoperatória com potenciais evocados somatossensoriais e motores não estava disponível na instituição.

### Análise Estatística

Os dados quantitativos foram expressos como média e desvio-padrão e submetidos ao teste de normalidade de Kolmogorov-Smirnov. A comparação entre grupos utilizou o teste *t* de Student ou análise de variância ( *analysis of variance* , ANOVA, em inglês)/Bonferroni (dados paramétricos), e os testes de Mann-Whitney ou de Kruskal-Wallis/Dunn (dados não paramétricos). A análise intragrupo foi realizada por meio do teste t pareado (dados paramétricos) ou teste de Wilcoxon (dados não paramétricos). Os dados categóricos foram expressos como frequências absolutas e percentuais e associados por meio do teste exato de Fisher ou do Qui quadrado de Pearson. Todas as análises foram realizadas no programa IBM SPSS Statistics for Windows (IBM Corp.), versão 20.0, com adoção do IC95%.

## Resultados

De uma população inicial de 168 pacientes (221 espirometrias), após a aplicação dos critérios de inclusão e exclusão, chegou-se a uma amostra de 9 pacientes elegíveis e avaliados por meio de espirometria pós-operatória.

A análise dos valores pré-operatórios das variáveis clínicas e radiográficas, bem como do teste de função pulmonar dos pacientes com escoliose sindrômica com e sem acompanhamento, revelou a ausência de diferenças significativas entre eles ( Tabela 1 ).

**Tabela 1:** Comparação entre variáveis clínicas e radiográficas pré-operatórias de pacientes com e sem acompanhamento

	Acompanhamento		
	Sim	Não	Valor de *p*
Idade média no último TFP antes da cirurgia (anos)	16,2 ± 3,9	18,6 ± 9,9	0,50 ^a^
Sexo (feminino/masculino)	5/4	7/4	1,00 ^b^
Altura média corrigida pela envergadura (cm)	142,3 ± 56,3	151,7 ± 17,7	0,60 ^a^
Neurofibromatose tipo 1/Síndrome de Marfan	5/4	8/3	1,00 ^b^
Índice de Massa Corporal médio (kg/m ^2^ )	16,0 ± 1,8	16,3 ± 2,5	0,74 ^a^
FVC média (L)	1,5 ± 0,7	1,8 ± 1,2	0,39 ^a^
%	41,4 ± 12,9	57,1 ± 25,7	0,11 ^a^
FEV1 médio (L)	1,2 ± 0,6	1,5 ± 1,0	0,42 ^a^
%	39,7 ± 14,9	54,0 ± 25,4	0,15 ^a^
Média do ângulo de Cobb da curva torácica principal (°)	71,8 ± 13,3	92,2 ± 21,5	0,06 ^a^

**Abreviaturas:** FVC, *forced vital capacity* (capacidade vital forçada); FEV1, *forced expiratory volume in 1 second* (volume expiratório forçado em 1 segundo); TFP, teste de função pulmonar.

**Notas:**^a^ Teste *t* de Student; ^b^ teste exato de Fisher; * *p* < 0,05.

Além disso, verificou-se a ausência de diferença significativa entre os grupos em relação às variáveis idade à cirurgia e ao último acompanhamento, sexo, altura pré-operatória corrigida pela envergadura, IMC pré-operatório, níveis de fusão, perda sanguínea estimada e tempo de acompanhamento. Esse valor foi maior nos pacientes com síndrome de Marfan, aproximadamente 16 anos, mas foi superior a 10 anos em ambos os grupos ( Tabela 2 ).

**Tabela 2:** Comparação entre parâmetros clínicos e radiográficos de acordo com a etiologia da escoliose sindrômica

	Síndrome de Marfan	NF1	Valor de *p*
**Idade média à cirurgia (anos)**	15,2 ± 4,4	17,5 ± 3,4	0,42 ^a^
**Idade média ao último acompanhamento (anos)**	32,7 ± 8,3	31,4 ± 4,7	0,78 ^a^
**Sexo: n(%)**			0,52 ^b^
Masculino	2 (40,0%)	3 (75,0%)	
Feminino	3 (60,0%)	1 (25,0%)	
**Altura média pré-operatória corrigida pela envergadura (cm)**	121,3 ± 69,5	168,5 ± 19,3	0,23 ^a^
**Índice de Massa Corporal médio pré-operatório**	16,5 ± 2,0	15,4 ± 1,7	0,43 ^a^
**Média dos níveis fundidos**	11,8 ± 1,6	11,7 ± 1,7	0,97 ^a^
**Média de perda de sangue estimada (mL)**	2.624,2 ± 2081,9	2.255,5 ± 723,5	0,75 ^a^
**Duração média da cirurgia (minutos)**	577,0 ± 178,1	598,7 ± 167,4	0,86 ^a^
**FVC média pré-operatória: n (%)**			
≤ 50%	3 (60,0%)	4 (100,0%)	0,44 ^b^
50–65%	2 (40,0%)	0 (0,0%)	
65–80%	0 (0,0%)	0 (0,0%)	
80–100%	0 (0,0%)	0 (0,0%)	
**FEV1 médio pré-operatório: n (%)**			
< 35%	1 (20,0%)	2 (50,0%)	0,52 ^b^
35–49%	2 (40,0%)	2 (50,0%)	
50–59%	1 (20,0%)	0 (0,0%)	
60–69%	1 (20,0%)	0 (0,0%)	
> 70%	1 (20,0%)	0 (0,0%)	
**Acompanhamento médio (anos)**	15,9 ± 4,5	12,4 ± 1,9 PM	0,20 ^a^

**Abreviaturas:** FVC, *forced vital capacity* (capacidade vital forçada); FEV1, *forced expiratory volume in 1 second* (volume expiratório forçado em 1 segundo); NF1, neurofibromatose tipo 1.

**Notas:**^a^ Teste *t* de Student; ^b^ teste exato de Fisher; * *p* < 0,05.

Em relação aos parâmetros de função pulmonar pré-operatória entre os grupos, verificou-se que metade dos pacientes com síndrome de Marfan apresentavam comprometimento pulmonar grave, enquanto todos os portadores de neurofibromatose apresentavam comprometimento pulmonar grave. Vale ressaltar que não houve diferenças entre os grupos em relação a essas variáveis ( Tabela 2 ).

Ao comparar os valores radiográficos pré e pós-operatórios entre os grupos, observou-se redução significativa ( *p* < 0,05) na magnitude do ângulo de Cobb da curva torácica principal após a cirurgia nos pacientes com síndrome de Marfan. Entretanto, não houve diferença nessa variável entre os grupos, assim como nos valores pré e pós-operatórios da cifose torácica ( Tabela 3 ).

**Tabela 3:** Acometimento de parâmetros pulmonares e radiográficos em pacientes com escoliose secundária à síndrome de Marfan e NF1 distrófica

	Síndrome de Marfan	NF1 distrófica	Valor de *p*
**FVC média (L)**			
Pré-operatória	3,2 ± 1,2	3,9 ± 0,9	0,37 ^a^
Pós-operatória	2,3 ± 0,7	2,3 ± 1,7	0,96 ^a^
**Valor de** ***p***	0,29 ^b^	0,09 ^b^	
Pós–pré-operatória	-0,9 ± 1,7	-1,6 ± 1,26	0,55 ^a^
**FEV1 médio (L)**			
Pré-operatório	1,3 ± 0,9	1,2 ± 0,3	0,87 ^a^
Pós-operatório	1,9 ± 0,7	1,9 ± 1,4	0,98 ^a^
**Valor de** ***p***	0,23 ^b^	0,36 ^b^	
Pós–pré-operatório	0,7 ± 1,1	0,7 ± 1,4	0,95 ^a^
**FVC média (%)**			
Pré-operatória	44,9 ± 16,6	37,0 ± 5,3	0,40 ^a^
Pós-operatória	39,6 ± 23,4	35,0 ± 12,2	0,73 ^a^
**Valor de** ***p***	0,67 ^b^	0,71 ^b^	
Pós–pré-operatória	-5,3 ± 25,6	-2,0 ± 9,9	0,82 ^a^
**FEV1 médio (%)**			
Pré-operatório	43,4 ± 19,4	35,0 ± 6,7	0,44 ^a^
Pós-operatório	53,2 ± 14,3	34,7 ± 11,3	0,07 ^a^
**Valor de** ***p***	0,20 ^b^	0,95 ^b^	
Pós–pré-operatório	9,8 ± 14,5	-0,3 ± 8,4	0,26 ^a^
**Média do ângulo de Cobb da curva torácica principal (°)**			
Pré-operatório	74,0 ± 16,3	69,0 ± 10,0	0,61 ^a^
Pós-operatório	46,2 ± 18,0	68,2 ± 21,2	0,13 ^a^
**Valor de** ***p***	** 0,04 ^b^**	0,96 ^b^	
Pós–pré-operatório	-27,8 ± 20,5	-0,7 ± 26,9	0,13 ^a^
**Cifose torácica média (T5–T12) (°)**			
Pré-operatória	30,0 ± 14,1	–	–
Pós-operatória	56,2 ± 28,2	–	–
**Valor de** ***p***	0,18 ^b^	–	–
Pós–pré-operatória	41,0 ± 17,0	–	–

**Abreviaturas:** FVC, *forced vital capacity* (capacidade vital forçada); FEV1, *forced expiratory volume in 1 second* (volume expiratório forçado em 1 segundo); NF1, neurofibromatose tipo 1.

**Notas:**^a^ Teste *t* de Student; ^b^ teste *t* pareado; * *p* < 0,05.

Ao analisar os valores percentuais e absolutos previstos de FEV1 e da FVC pré e pós-operatórios, não foram encontradas diferenças significativas dentro do mesmo grupo ou entre eles durante o último acompanhamento ( Tabela 3 ).

A avaliação da razão de chances entre a etiologia da escoliose sindrômica e complicações pulmonares em pacientes submetidos à cirurgia de abordagem dupla mostrou a ausência de maior chance de complicações pulmonares de uma síndrome em relação à outra ( Tabela 4 ).

**Tabela 4:** Análise da razão de chances entre a presença de complicações pulmonares pós-operatórias e a etiologia da escoliose

	Complicações pulmonares	Valor de *p*	Razão de chances (IC95%)
	Sim: n (%)			Não: n (%)
**Etiologia da escoliose**			0,20	
Síndrome de Marfan	1 (25,0%)	4 (80,0%)		12,0 (0,5–28,1)
Neurofibromatose tipo 1 distrófica	3 (75,0%)	1 (20,0%)		

**Notas:** Teste exato de Fisher; * *p* < 0,05.

Os três pacientes com NF1 submetidos à toracotomia seguida de artrodese posterior apresentaram as seguintes complicações: atelectasia, pneumotórax, hemotórax e espessamento fibrótico da pleura em um paciente; pneumonia e insuficiência respiratória no segundo; e pneumonia no último. O paciente com síndrome de Marfan foi submetido à mesma abordagem cirúrgica e apresentou atelectasia como complicação pulmonar.

## Discussão

Este estudo avaliou pacientes com diagnóstico sindrômico definido e divididos em dois grupos, ou seja, Marfan e NF1 distrófica, com tempo de acompanhamento superior a 10 anos. No primeiro grupo, os pacientes apresentavam curvas rígidas (flexibilidade < 30%) e, no segundo, além da rigidez, a presença de características distróficas/displásicas. Isso influenciou a escolha da abordagem combinada.

Os casos cirúrgicos de pacientes com escoliose por síndrome de Marfan geralmente envolvem níveis mais fusionados. Nestes casos, a liberação anterior, por meio da remoção dos discos intervertebrais ao redor do ápice da deformidade, aumenta a flexibilidade da curva, reduzindo as forças de cisalhamento entre a interface dos implantes e as estruturas ósseas de fixação. 2

Em nosso estudo, o número médio de níveis combinados entre os dois grupos (Marfan e NF1) não diferiu, sendo de 11,80 ± 1,6 e 11,75 ± 1,7, respectivamente, maior do que o utilizado em casos de artrodese seletiva, em que o nível máximo fica em torno de 8 a 10 em curvas torácicas.

Pacientes com escoliose menor que 55° já podem apresentar declínio da FVC. Vale ressaltar que quanto mais cefálico o ápice da deformidade e menor a cifose do paciente, maior o comprometimento da função pulmonar. 4

Na presente série, os pacientes apresentaram cifose torácica média de 30 ± 14,1° e ângulo de Cobb da curva torácica principal de 74 ± 16,3° antes da cirurgia. Em relação ao comprometimento pulmonar pré-operatório, observou-se que cerca de metade dos pacientes apresentava comprometimento grave, com porcentagens de FEV1 menor que 50%, e a maioria tinha FVC ≤ 50%.

Um estudo que avaliou a função pulmonar pré-operatória de pacientes com escoliose secundária à síndrome de Marfan não observou correlação entre os valores pré-operatórios da cifose torácica (Cobb de T5–T12) e da magnitude da curva (Cobb da curva torácica em plano coronal) e os valores de FVC e FEV1. Neste estudo, as porcentagens médias de FVC e FEV1 foram de 70,12 ± 22,40 e 67,07 ± 20,213%, respectivamente. 3

Em nossa série, o grupo de pacientes com síndrome de Marfan apresentou redução significativa na magnitude da curva (74,0→46,2°) após o procedimento cirúrgico. Em relação à avaliação da função pulmonar, não houve variação significativa nos valores percentuais previstos de FVC (-5,28 ± 25,65%) e FEV1 (9,82 ± 14,46%) entre o período pré-operatório e o último acompanhamento.

Em uma coorte de pacientes com escoliose secundária à síndrome de Marfan submetidos à cirurgia por dupla abordagem, as complicações cirúrgicas encontradas foram afrouxamento do parafuso em dois pacientes, quebra da haste em um, e quilotórax com hemotórax em outro paciente submetido à toracoscopia. 2

Ao avaliar as complicações em pacientes com síndrome de Marfan, observamos um caso de atelectasia, tratado com sucesso com antibióticos e fisioterapia respiratória. Não houve complicações relacionadas ao implante.

O outro grupo estudado, dos pacientes com escoliose secundária à NF1 com padrão distrófico, era caracterizado por curva torácica única, rígida, curta e acentuada; diminuição da largura das costelas ( *rib pencilling* ); cunhagem de um ou mais corpos vertebrais; massas em tecidos para- ou intravertebrais; rotação acentuada no ápice da deformidade; pedículos displásicos; e/ou aumento do canal vertebral e do forame. Segundo alguns autores, na presença de três ou mais dessas características, a chance média de progressão da deformidade é de 85%, com risco de comprometimento da função pulmonar. 5

Em nosso estudo, os pacientes diagnosticados com NF1 apresentavam escoliose com curva torácica rígida e curta (Cobb pré-operatório: 69 ± 10°), associada a pedículos displásicos e alterações nos arcos costais (diminuição da largura das costelas). Estas anomalias foram associadas ao comprometimento pulmonar pré-operatório grave, com porcentagens de FVC e FEV1 abaixo de 50%.

Alguns autores sugerem que, em casos distróficos, a cirurgia deve ser realizada o mais breve possível devido à rápida progressão da curva. A realização da artrodese em pacientes mais jovens não necessariamente resultaria em perda da altura final, pois o número de níveis fundidos seria menor. 6

Em nosso estudo, os pacientes foram operados, em média, após o estirão de crescimento e próximo ao final da adolescência (cerca de 17 anos) com uma altura corrigida pré-operatória de 168,50 ± 19,3 cm e uma média de cerca de 12 níveis fundidos após a cirurgia.

Um estudo que analisou os resultados da cirurgia combinada à toracotomia em pacientes com escoliose distrófica de início precoce (< 10 anos de idade) mostrou redução na magnitude da curva (71,2→23,5°) e alterações nos valores pré- e pós-operatórios de FVC (1,4→2,3 L) e %FVC (75,0→74%). 5

Não observamos melhora significativa no ângulo de Cobb da curva torácica principal após a cirurgia ou na variação (pós-operatório – pré-operatório) dos parâmetros de função pulmonar: FVC (-1,57 ± 1,26 L), FEV1 (0,76 ± 1,40 L), e porcentagens (FVC: -2,00 ± 9,94%; e FEV1: -0,28 ± 8,41%).

Ao avaliar os resultados de diferentes estudos em pacientes com escoliose secundária à NF1 com padrão distrófico, Jia et al. mostraram que houve redução significativa do ângulo de Cobb da curva torácica principal em pacientes submetidos à artrodese combinada e artrodese posterior isolada, sem diferença entre os dois grupos. Segundo estes autores, as duas abordagens têm eficácia semelhante, estabilidade e segurança no acompanhamento pós-operatório desses pacientes. 13

Em relação às complicações após a abordagem anterior e posterior desses pacientes, alguns autores relataram que cerca de 28,5% deles evoluíram com complicações, tais como mau posicionamento e soltura dos implantes, fístula dural, pseudoartrose, atelectasia, úlcera por pressão, infecção superficial, paraplegia e paraparesia transitórias e fenômeno de *crankshaft* , entre outras. Os autores ressaltam que a proporção de falha de instrumentação ou pseudoartrose foi de 8,3%. 13

Em nosso estudo, três pacientes com NF1 submetidos à toracotomia seguida de artrodese posterior apresentaram as seguintes complicações: atelectasia, pneumotórax, hemotórax e espessamento fibrótico da pleura em um indivíduo tratado com drenagem torácica e fisioterapia pulmonar; pneumonia e insuficiência respiratória em outro paciente, que foi submetido a intubação orotraqueal e antibioticoterapia intravenosa; e pneumonia no último paciente. Todos eles evoluíram bem clinicamente. Deve-se notar que nenhum dos pacientes desenvolveu complicações relacionadas aos implantes.

Ao comparar os dois grupos de pacientes do presente estudo, observou-se que, embora os pacientes com NF1 apresentassem valores menores (pior comprometimento) dos parâmetros de função pulmonar antes da cirurgia e no último acompanhamento em comparação ao grupo com síndrome de Marfan, essa diferença não foi significativa. Além disso, não houve maior chance de complicações pulmonares entre uma etiologia e outra utilizando a mesma abordagem cirúrgica.

Nosso estudo tem alguns pontos fortes, como o fato de ser um dos poucos a apresentar parâmetros de função pulmonar pré e pós-operatórios em pacientes com escoliose sindrômica com acompanhamento longo (maior que 10 anos). No entanto, deve-se ter cautela na interpretação dos resultados e na sua validação externa devido às limitações inerentes a esta pesquisa.

As limitações podem ser atribuídas à natureza retrospectiva do estudo; sua realização em um único centro; a amostra pequena e a ausência de avaliação do efeito apenas da fusão espinhal posterior, tração esquelética, osteotomias vertebrais e toracoplastia na função pulmonar.

## Conclusão

Ao avaliar o efeito da fusão espinhal combinada (anterior e posterior) na função pulmonar em pacientes com escoliose sindrômica (síndrome de Marfan e NF1) no acompanhamento em longo prazo (> 10 anos), verificou-se que não houve diferença nos valores percentuais e absolutos previstos de FVC e FEV1 entre os grupos no último acompanhamento. Além disso, os valores de função pulmonar não apresentaram alterações significativas na última avaliação pós-operatória em comparação ao período pré-operatório.
